# PET/MRI in Oncology: Real-World Recommendations

**DOI:** 10.3390/cancers18213546

**Published:** 2026-11-04

**Authors:** Ana María García-Vicente, Lina García-Cañamaque, José Ramón García-Garzón, Montserrat Cortés-Romera, David Durany-Lara, Miguel Sebastián Casallas-Cepeda, Stefanía Aida Guzmán-Ortiz, Jaime Andrés Bonilla-Plaza, Edel Noriega-Álvarez

**Affiliations:** 1 Nuclear Medicine Department, University Hospital of Toledo, 45007 Toledo, Spain; 2 Artificial Intelligence and Molecular Imaging Group, Instituto de Investigación Sanitaria de Castilla La Mancha (IDISCAM), 45071 Toledo, Spain; edeln@sescam.jccm.es; 3 Nuclear Medicine Department, Grupo Universitario HM Hospitales, 28050 Madrid, Spain; 4 Positron Emission Tomography Center Cetir Ascires, Viladomat, 08021 Barcelona, Spain; 5 Nuclear Medicine Department, Institute of Diagnostic Imaging (IDI), Hospital Universitario de Bellvitge, L’Hospitalet de Llobregat, 08907 Barcelona, Spain; 6 Department of Radiodiagnostics, Institute of Diagnostic Imaging (IDI), Germans Trias i Pujol University Hospital, 08916 Badalona, Spain; david.durany.idi@gencat.cat; 7 Nuclear Medicine Department, University Hospital of Guadalajara, 45007 Toledo, Spain

**Keywords:** PET/MRI, hybrid imaging, molecular imaging, oncology, tumor staging, treatment response assessment, multiparametric imaging, radiomics

## Abstract

Positron emission tomography/magnetic resonance imaging (PET/MRI) has represented a paradigm shift in medical imaging, and although its use has not yet been fully incorporated into all clinical guidelines, scientific evidence supports its ability to provide a highly accurate simultaneous metabolic and anatomical assessment, which is particularly useful for staging, treatment planning, and evaluating treatment response in cancer patients. The limited availability of the technique and the demands it places on resources and coordination suggest that its initial use should focus on those applications that can provide the greatest benefit to patients, especially within the indications related to oncological processes, where PET and MRI have a defined and complementary role. This review focuses specifically on some of these conditions.

## Simple Summary

Positron emission tomography combined with magnetic resonance imaging, known as PET/MRI, merges a scan that shows how active a tumor is with a scan that shows detailed anatomy, in a single examination. Although this combined technique is not yet part of every clinical guideline, growing evidence shows it can give more accurate information than either scan used alone, helping doctors detect tumors, plan treatment, and monitor how well a treatment is working. Given PET/MRI machines are scarce and require substantial resources and coordination between specialists, its use currently makes most sense for the cancers in which it offers the clearest advantage.

## 1. Introduction

Positron emission tomography/magnetic resonance imaging (PET/MRI) combines the molecular and metabolic information provided by PET with the superior soft-tissue contrast and multiparametric capabilities of MRI in a single examination, offering an attractive alternative to sequential PET/CT and MRI studies. Its clinical use has expanded across oncology, supporting primary tumor characterization, locoregional and distant staging, treatment response assessment, and the development of imaging and radiomic biomarkers, while reducing radiation exposure and improving patient comfort compared with combined PET/CT and MRI protocols performed separately.

In Spain, there are 4 PET/MRI units used for clinical care, 2 of which have more than 7 years of use. Based on the experience gained by these centers, this review has focused on oncological conditions in which PET/MRI has demonstrated its potential compared to other diagnostic techniques. For PET/MRI results, descriptions included studies were selected according to predefined inclusion criteria: studies report patients of any age group who underwent PET/MRI on selected pathologies using hybrid scans. No temporary restriction was applied. Editorials, commentaries, conference abstracts, or case reports/series with fewer than five cases were excluded.

This review summarizes the current evidence and Spanish center experience on how combined PET and MRI scanning can help in the diagnosis, staging, and follow-up of prostate, gynecological, liver and pancreatic, brain, bone and soft-tissue, rectal, and breast cancers, highlighting the situations in which it adds the most value for patients.

## 2. Prostate Cancer

In prostate cancer (PC), multiparametric MRI (mpMRI) is the gold standard for evaluating the primary tumor and its local extent, while PET with ligands targeting prostate-specific membrane antigen (PSMA) clearly outperforms conventional imaging techniques (CIT), such as computed tomography (CT) and bone scintigraphy, in all clinical contexts of the disease.

Regarding the evaluation and interpretation of PET images, the use of standardized criteria—such as those derived from the PRIMARY trial for the exclusive assessment of the primary tumor, and the more general PROMISE (Prostate Cancer Molecular Imaging Standardized Evaluation) and PSMA-RADS (Prostate-Specific Membrane Antigen Reporting and Data System) allows for a more reproducible assessment of PSMA expression, which is useful in characterizing lesions and, therefore, in prognostic stratification as well as in treatment planning, enabling better comparison between follow-up studies [1,2,3].

The interpretation of mpMRI, in terms of primary tumor assessment, is governed by the PI-RADS 2.1 system (Prostate Imaging Reporting and Data System) [4], whereas the detection and characterization of local recurrence after prostatectomy or radiation therapy, or whole-disease assessment on whole-body MRI, has been standardized using the PI-RR (Prostate Imaging Recurrence Reporting) system [5,6] and MET-RADS-P (Metastasis Reporting and Data System for Prostate Cancer) system, respectively [7].

The advantages of mpMRI in assessing and defining the extent of the primary tumor and of PSMA-PET in the initial regional and distant staging of high-risk patients, with diagnostic and therapeutic impact in approximately one-third of patients, justify the added value associated with the combined use of both techniques, especially in patients who are candidates for curative local treatments [8,9,10]. Furthermore, in the context of biochemical recurrence, PSMA-PET is the technique of choice, enabling the detection of oligometastatic disease, which allows for the individualization of targeted salvage therapies and helps avoid or delay unnecessary systemic treatments [11,12]. Based on these premises, the clinical scenarios in which patients may benefit from the hybrid PET/MRI technique are defined below.

### 2.1. Characterization of Indeterminate Prostate Lesions and Biopsy Guidance

**Figure 1:**
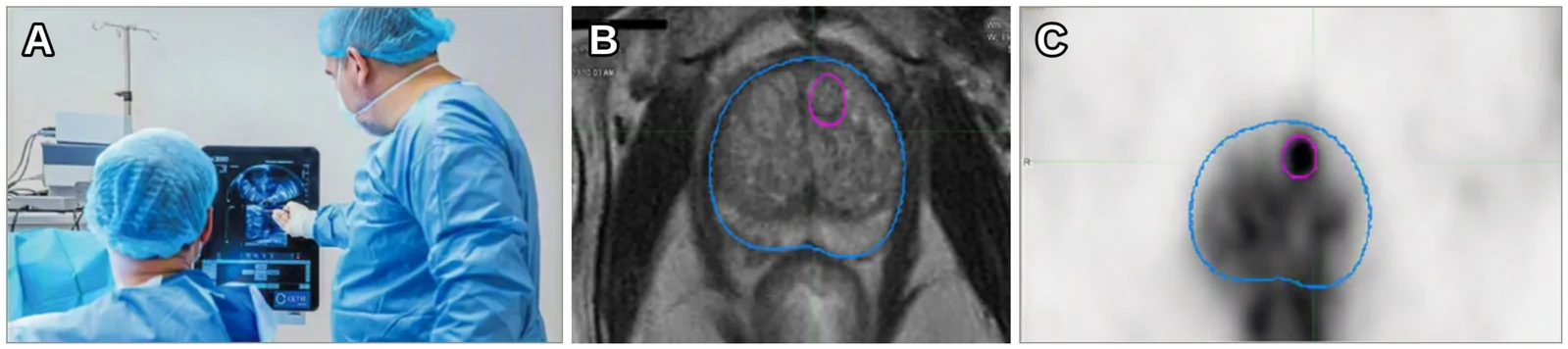
Planning for a transperineal prostate biopsy (**A**) guided by fusion of PSMA PET/MRI (**B**) with transrectal ultrasound. The mpMRI showed a PI-RADS 3 lesion, while the PET scan (**C**) showed a focal PSMA uptake (pink circle) on prostate (blue circle) classified as PRIMARY ≥ 3. Final diagnosis: Gleason 4 + 3 prostate adenocarcinoma.

PSMA uptake (PRIMARY ≥ 3) allows for a significantly more accurate categorization of indeterminate lesions (PI-RADS 3) on mpMRI. Using specific fusion software and PET/MRI-guided biopsy with PSMA, a PC detection rate of 80% has been determined, with greater efficacy in the diagnosis of significant prostate cancer (sPC) [13]. Furthermore, combining PET with mpMRI increases detection accuracy to 88%, supporting the use of PET/MRI as an integrated technique [14]. Emmet et al. [15] demonstrate the superiority of the combined technique, with sensitivity values for mpMRI, PSMA-PET, and the PET/MRI combination of 83%, 90%, and 97%, respectively, and a negative predictive value (NPV) for PET/MRI of 91% compared to 72% for mpMRI. Therefore, a PI-RADS 3 lesion identified on mpMRI that exhibits significant PSMA uptake (PRIMARY ≥ 3) indicates the need for a PET/MRI-guided biopsy. This metabolic correlation offers high sensitivity, making it possible to avoid unnecessary biopsies in those PI-RADS 3 lesions that do not show pathological uptake (PRIMARY 1–2) Figure 1.

On the other hand, the use of PET/MRI appears particularly relevant in patients with previous negative biopsies, especially in lesions classified as PIRADS 4 and 5, where triage using mpMRI improves diagnostic accuracy in detecting PC from 48% to 93% of patients, and specifically in detecting sPC (26% vs. 38%), compared with systematic biopsy. Another area of study, given the synergy of PET/MRI with PSMA, is the optimized intraprostatic localization of dominant tumor foci (those with the highest PET activity and PIRADS 4–5 classification) to facilitate targeted radical treatment strategies [16,17].

### 2.2. Assessment of Locoregional Disease

**Figure 2:**
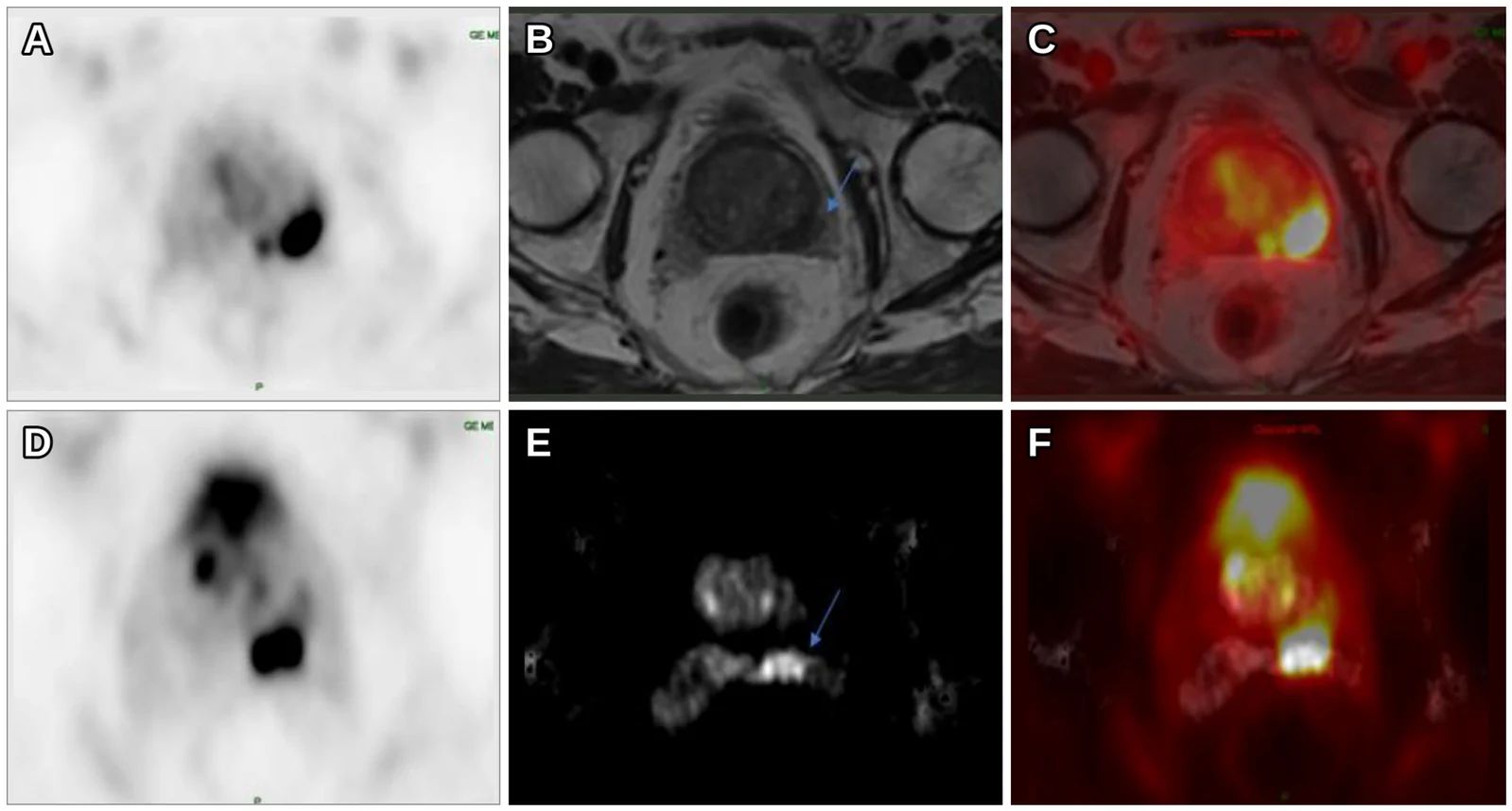
T3 staging using selective PSMA-guided PET/MRI. Top row. Example of ECE with a PSMA-PET-enhanced lesion in the peripheral zone of the left lobe, along with a smaller medial lesion (**A**); the exact location of the larger lesion is defined on a T2-weighted MRI image ((**B**), arrow), while the other is undetectable on this image. Co-registered image (**C**). Bottom row. Example of SVI. A lesion showing uptake on the right side that is consistent on PSMA-PET (**D**) and shows diffusion restriction on MRI ((**E**), arrow). Co-registered image (**F**).

In patients with an initial diagnosis of PC, in the context of T-stage classification, mpMRI is the standard of care for assessing extracapsular extension (ECE) and seminal vesicle invasion (SVI)—factors that determine classification as T3a and T3b, respectively, and critically influence the surgical strategy. However, the sensitivity of mpMRI for determining ECE is moderate, and interobserver variability exists [18]. Although the sensitivity and specificity of PSMA-PET and mpMRI in detecting ECE and SVI are comparable, mpMRI retains a slight advantage in sensitivity for SVI [19]. Therefore, the combined use of PSMA-PET/MRI improves the detection of tumor extension outside the prostate, with greater sensitivity in determining ECE and neurovascular invasion (87% vs. 57%) as well as SVI and regional extension, when compared to mpMRI alone [20,21,22] Figure 2.

**Figure 3:**
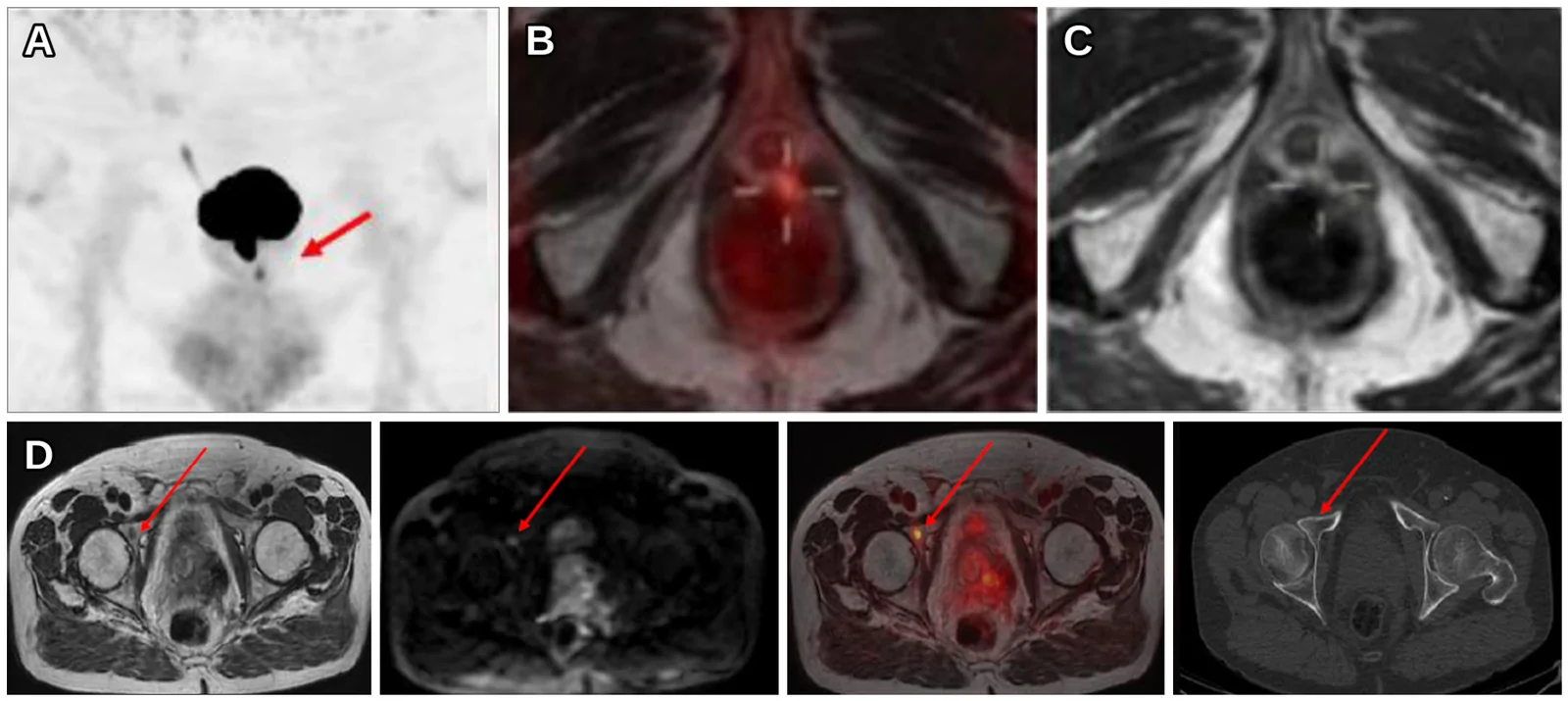
Top row: Local recurrence after prostatectomy (arrow) detected by PSMA-PET/MRI. (**A**) PSMA-PET MIP (maximum intensity projection), location defined by fusion with MRI (T2) (**B**); no suspicious lesions are visible on the isolated T2-weighted MRI (**C**) (MRI false negative). Bottom row (**D**): distant bone recurrence detected by PSMA-PET/MRI (arrow). From left to right: axial T1-weighted MRI, diffusion-weighted MRI, PET/MRI fusion, and bone-window CT. Bone metastasis in the right acetabulum was not detected on CT but was confirmed on MRI (T1 and diffusion) and showed increased uptake on PET. Benign lesion not detected on additional CT, confirmed by the MRI component of the hybrid scan.

Regarding the assessment of local recurrence after radical prostatectomy, the use of PET and MRI plays a complementary role in selected patients—for example, those with prostate-specific antigen (PSA) < 1 ng/mL—improving the detection of local disease by 14% compared to CIT [9] Figure 3. Furthermore, in the detection of active disease following prostate radiation therapy, PSMA-PET can provide significant support to MRI, since with the latter, the diagnosis of local recurrence is more complex due to atrophy, glandular fibrosis, fatty replacement, and thinning of the fasciae; in this context, the dynamic contrast-enhanced (DCE) sequence is essential [8,23].

In the detection of lymphadenopathy, the diffusion sequence on MRI adds diagnostic certainty to PET compared to CT, especially if the size is more than one centimeter. Conversely, an absence of PET activity in a lymph node that shows restricted diffusion on MRI increases the specificity of the latter, indicating the possible inflammatory nature of the lesion. Thus, a PET-hyperuptake lymph node that shows diffusion restriction on MRI is suggestive of malignancy. Furthermore, in inflammatory lymph nodes, contrast enhancement is less intense and slower (gradual enhancement pattern) compared to the rapid and intense enhancement seen in metastatic lymphadenopathy.

### 2.3. Assessment of Distant Disease

Regarding bone metastases, the high sensitivity of PSMA-PET uptake presents a diagnostic challenge in cases where there is no corresponding finding on the CT component; consequently, in many cases, these are classified as indeterminate (PSMA-RADS 3) and require further confirmation [3]. This is particularly relevant if the bone lesions are isolated or predominantly costal. In these cases, MRI provides additional certainty to the PET scan, helping to avoid potential false positives [24].

In addition, PET/MRI allows for the detection of early bone marrow infiltration using STIR (short tau inversion recovery) and diffusion sequences, before morphological changes are visible on CT and even prior to the appearance of signal changes on T2-weighted sequences, thereby avoiding false positives due to nonspecific uptake on PSMA-PET caused by benign lesions such as hemangiomas or dysplasia [25] Figure 3.

### 2.4. Imaging Biomarkers and Radiomics

Semi-quantitative analyses of various imaging biomarkers allow for the characterization of tumor heterogeneity by classifying lesions; ADC (apparent diffusion coefficient) maps have been shown to perform better than T2-weighted images, thereby reducing the subjectivity of visual analysis. In addition, there is a need to explore the radiomic data obtained from PSMA-guided PET/MRI studies to validate different machine learning models for assessing the risk of developing PC, particularly clinically significant sPC, determining the degree of local invasion, and even predicting patient prognosis [10,26,27,28]. Furthermore, analyzing the textural characteristics of the primary tumor can optimize the prediction of ECE [17,29].

## 3. Gynecological Tumors

PET/MRI offers advantages both in detecting local spread in gynecological tumors and in evaluating lymph node and distant metastases.

### 3.1. Cervical Cancer

MRI is the standard technique for assessing local extent in both cervical cancer (cervical stroma, parametrium, vagina, bladder/rectum) and endometrial cancer (depth of myometrial invasion, cervical invasion, and extrauterine extension)—data that are essential for therapeutic decision-making [30]. In women of reproductive age, cyclical metabolic changes in the endometrium and ovaries, as well as urinary activity in the ureters, can mimic pathological activity if not properly contextualized [31,32,33]. Therefore, specific diffusion sequences can resolve equivocal findings on [^18^F]FDG scans, reinforcing the value of combined PET/MRI interpretation [31].

**Figure 4:**
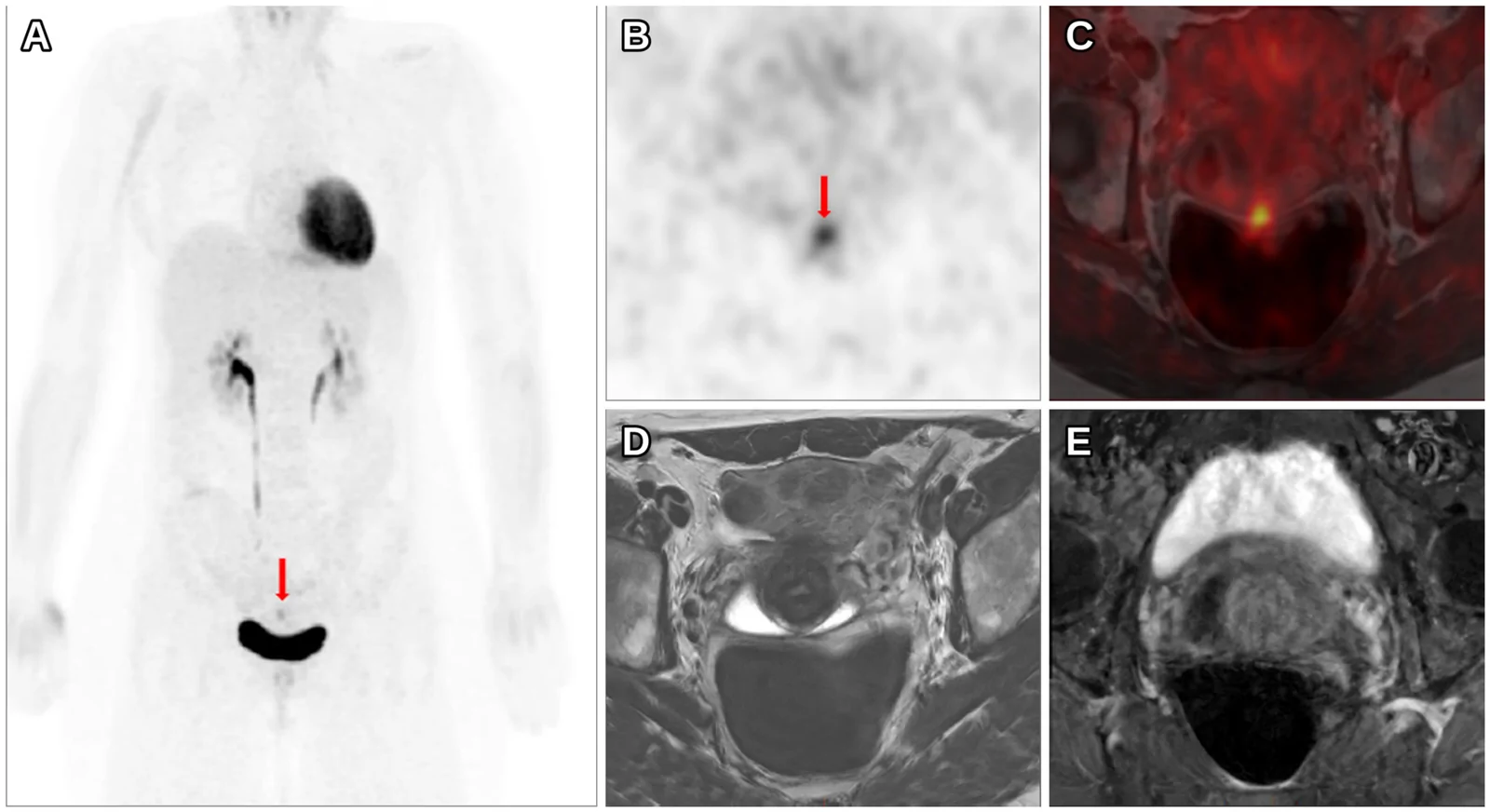
Initial staging of endocervical usual-type adenocarcinoma (T1a1 stage) with [^18^F]FDG PET/MRI. A small focal lesion is visible on the MIP image ((**A**), arrow) and the PET axial slice, ((**B**), arrow), located in the cervix on the co-registered PET/MRI-T1 image ((**C**), arrow). This uptake shows no pathological correlate on a high-resolution T2-weighted sequence (**D**) or on a fat-suppressed T1-Gd sequence (**E**). The patient was treated with conization and endocervical curettage, confirming cervical malignancy.

On the other hand, PET provides a more accurate assessment of lymph node and distant metastases; therefore, its use is indicated primarily for tumors with a higher probability of disease in these areas, such as inoperable cervical cancers —stages IB3 to IIA2 or higher according to the International Federation of Gynaecology and Obstetrics (FIGO. This aspect is key for selecting surgery versus chemotherapy/radiotherapy (Ch/RT) and for planning image-guided brachytherapy [34]. Figure 4 illustrates improved lesion detection based on the hybrid assessment. However, it should be noted that although [^18^F]FDG PET/MRI is a highly specific method for lymph node staging, its sensitivity is limited, especially for small lymph node metastases; therefore, it must be combined with sentinel lymph node (SLN) mapping [35].

In cases of suspected relapse, PET/MRI appears to be the optimal choice, particularly in cases with previously inconclusive CIT results [8,36]. The ESGO–ESTRO–ESP (European Society of Gynaecological Oncology—European Society for Radiotherapy and Oncology—European Society of Pathology) recommends pelvic MRI or expert ultrasound for local staging, and CT or PET/CT for extrapelvic spread, while considering PET/MRI at experienced centers when seeking to combine both assessments into a single examination with minimal radiation exposure [37].

In addition, PET/MRI is superior to other techniques in determining tumor burden, as the combination of diffusion restriction, gadolinium enhancement, and metabolic activity facilitates the accurate detection of peritoneal implants, increasing diagnostic sensitivity compared to MRI alone [33,34]. This approach allows for a more reliable estimation of the peritoneal cancer index (PCI), a key parameter for treatment planning, with significant added prognostic value.

Regarding response assessment, PET/MRI allows for distinguishing between tumor recurrence and changes induced by radiation therapy, such as fibrosis or edema consistent with an absence of diffusion restriction, as well as a lack of persistent [^18^F]FDG uptake several months after treatment [33]. Focusing on cervical cancer, Vojtíšek et al. [38] categorize three types of response on PET/MRI in patients treated with concurrent external beam ChRT and brachytherapy based on the RECIST 1.1 [39] and PERCIST [40] criteria, classifying: (i) tumor or lymph node response when both tests are negative at their respective sites, and (ii) persistence of disease when morphological or metabolic signs consistent with tumor activity are present. Dhesi and Weyl et al. [41,42] established a graded classification for both PET and MRI. Combined assessment of PET/MRI fusion using this scale increases the degree of interpretive certainty by 45%, with modifications in 31% of cases [41,42,43].

### 3.2. Ovarian Cancer

Although guidelines such as those from the National Comprehensive Cancer Network or the European Society of Medical Oncology prioritize PET/CT for the diagnosis of relapse—especially in cases with elevated CA-125 levels and negative or inconclusive CT scans—combining PET with MRI can broaden its scope of application, particularly in patients with suspected peritoneal carcinomatosis.

MRI has notable advantages over CT in detecting peritoneal dissemination, especially when lesions are small or located in “difficult” regions (intestinal serosa, right subdiaphragm, mesentery), with detection rates exceeding 80% when using diffusion-weighted sequences. On the other hand, although PET’s sensitivity does not exceed that of MRI, it has the advantage of a specificity close to 100%, so the diagnostic yield of PET/MRI will always be higher than that of PET/CT, improving the estimation of the peritoneal carcinomatosis index (PCI), which has a known impact on the selection of primary cytoreduction versus neoadjuvant therapy in one-third of the patients studied [44,45].

MRI is the most sensitive technique for detecting liver lesions, especially using dynamic and diffusion-weighted sequences. Its combination with PET increases diagnostic confidence compared to PET/CT, particularly for small lesions or those with low metabolism. Regarding the detection of pulmonary micronodules, the gold standard is inspiratory CT, although specific MRI sequences, with higher sensitivity than standard techniques, are currently being developed.

### 3.3. Imaging Biomarkers and Radiomics

In endometrial cancer, the SUV/ADC ratio has been defined as a biomarker of aggressiveness [46], predicting molecular alterations (p53abn, MMR-d) with an emerging role in risk stratification and personalized therapy [47].

In cervical cancer, compared with visual assessment, the incorporation of quantitative parameters—such as mean SUV, peak SUV, MRI-derived sphericity, and ADC values—increases sensitivity for detecting lymph node infiltration (from 31% to 79%), with a moderate decrease in specificity (from 98.3% to 75.6%). These results suggest that a multiparametric approach based on PET/MRI can substantially improve diagnostic accuracy and prognostic classification, outperforming both visual interpretation and independent evaluation using PET/CT or MRI [37,48,49,50].

## 4. Hepato-Bilio-Pancreatic Tumors

Integrated PET/MRI represents one of the most advanced imaging modalities available for evaluating the hepatobiliary and pancreatic systems. MRI’s ability to characterize soft tissues and assess hepatic infiltration or locoregional spread, combined with the metabolic information from the rest of the body provided by PET, can significantly improve overall staging in these patients.

### 4.1. Hepatocellular Carcinoma

In hepatocellular carcinoma (HCC), [^18^F]FDG PET/MRI can improve overall staging by detecting extrahepatic disease in high-grade tumors; furthermore, it provides prognostic value, since the degree of uptake is associated with microvascular invasion and cytokeratin 19 expression, crucial factors in determining the risk of recurrence after liver transplantation [51,52]. Vermersch M. et al. [53] reported that the sensitivity of PET/MRI in detecting metastases—both in terms of lesions and patients—was significantly higher than that of the whole-body CT/liver MRI combination (100% vs. 43%, *p* = 0.002; and 100% vs. 45%, *p* = 0.010, respectively).

For these reasons, PET/MRI could be considered for initial staging in patients who are candidates for local treatments (even helping to establish the dosimetry of the target lesion in patients eligible for ^90^Yttrium) or for transplantation [54]. Furthermore, PET/MRI can play a significant role in assessing the response to locoregional therapies, such as transarterial chemoembolization (TACE), radiofrequency ablation, or radioembolization with ^90^Yttrium, allowing for the simultaneous evaluation of morphological and metabolic changes in the tumor.

Although [^18^F]FDG is the most widely used radiopharmaceutical, other radiotracers with higher disease detection rates can be employed, especially in well-differentiated tumors, such as [^18^F]-fluorocholine (FCH) or even radiolabeled PSMA ligands, although there is currently no experience using the hybrid technique.

### 4.2. Cholangiocarcinoma

Performing PET with simultaneous acquisition alongside cholangiopancreatic MRI in the staging of cholangiocarcinoma allows for better visualization of the biliary tree and vascular invasion, in addition to facilitating the detection of small peritoneal and lymphatic metastases. This highlights the superiority of PET/MRI over other modalities such as PET, CT, and MRI, influencing clinical management in up to 30% of patients with untreated intrahepatic cholangiocarcinoma [8,55,56].

### 4.3. Pancreatic Cancer

PET/MRI can provide relevant additional information in pancreatic ductal adenocarcinoma, leading to changes in the treatment strategy in up to 49% of patients, primarily due to its greater sensitivity in detecting distant metastases and locoregional lymph node involvement [8,57,58].

### 4.4. Liver Metastases

**Figure 5:**
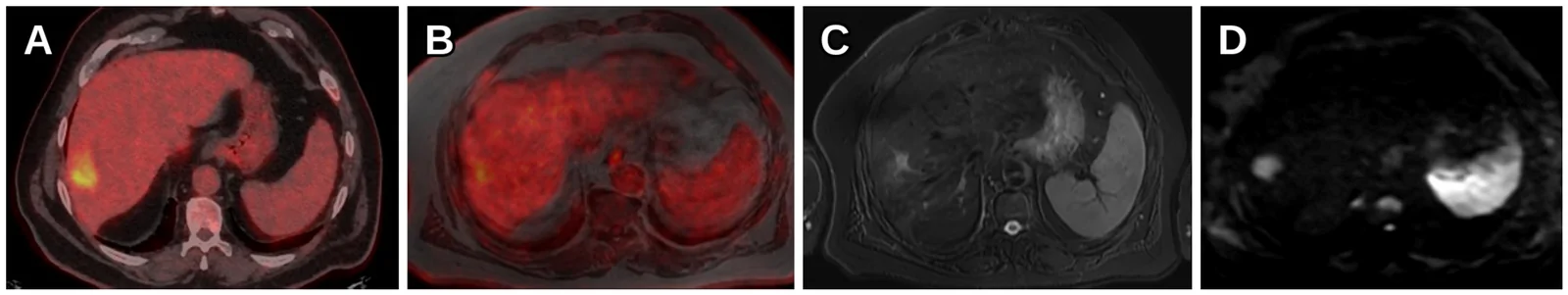
Liver metastases of a moderately differentiated colon adenocarcinoma (pT4pN1) who underwent surgery for intestinal obstruction. During follow-up, liver progression (single lesion) was diagnosed on a PET/CT axial slice (**A**). Chemotherapy was initiated, and on a preoperative PET/MRI scan, mild metabolism (**B**) was observed in a lesion with T2 signal abnormality (**C**) that restricted diffusion (**D**), consistent with tumor viability in a segment 7 liver metastasis, which was confirmed after surgery.

The combination of PET and MRI allows for the integration of metabolic and anatomical information using high-resolution sequences, thereby improving diagnostic performance. In a multicenter study, PET/MRI achieved a sensitivity of 95% and a specificity of 97% for the detection of liver metastases, characterizing 80% of lesions that were indeterminate on MRI and 91% of those that were indeterminate on PET [59]. Furthermore, PET/MRI has demonstrated advantages over other imaging modalities in detecting small and critically located liver metastases, such as subcapsular and perivascular lesions [60]. In the context of oligometastatic colorectal cancer, this results in a change in treatment strategy in approximately 19% of patients, as it allows for the identification of a higher tumor burden than initially suspected [61]. Likewise, PET/MRI has demonstrated superiority in detecting distant metastases from tumors of gastrointestinal origin by simultaneously identifying metabolic uptake on PET and morphological abnormalities on MRI, with a sensitivity of 97%, compared to 54% for CT [62]. In another study on the management of patients with colorectal cancer, the rate of change in management reached 21% [61], primarily involving upstaging that led to more aggressive surgeries or, alternatively, the decision to forgo surgical treatment and switch to systemic therapy. On the other hand, MRI detection of liver metastases during chemotherapy is improved over PET [63] Figure 5.

### 4.5. Neuroendocrine Tumors

**Figure 6:**
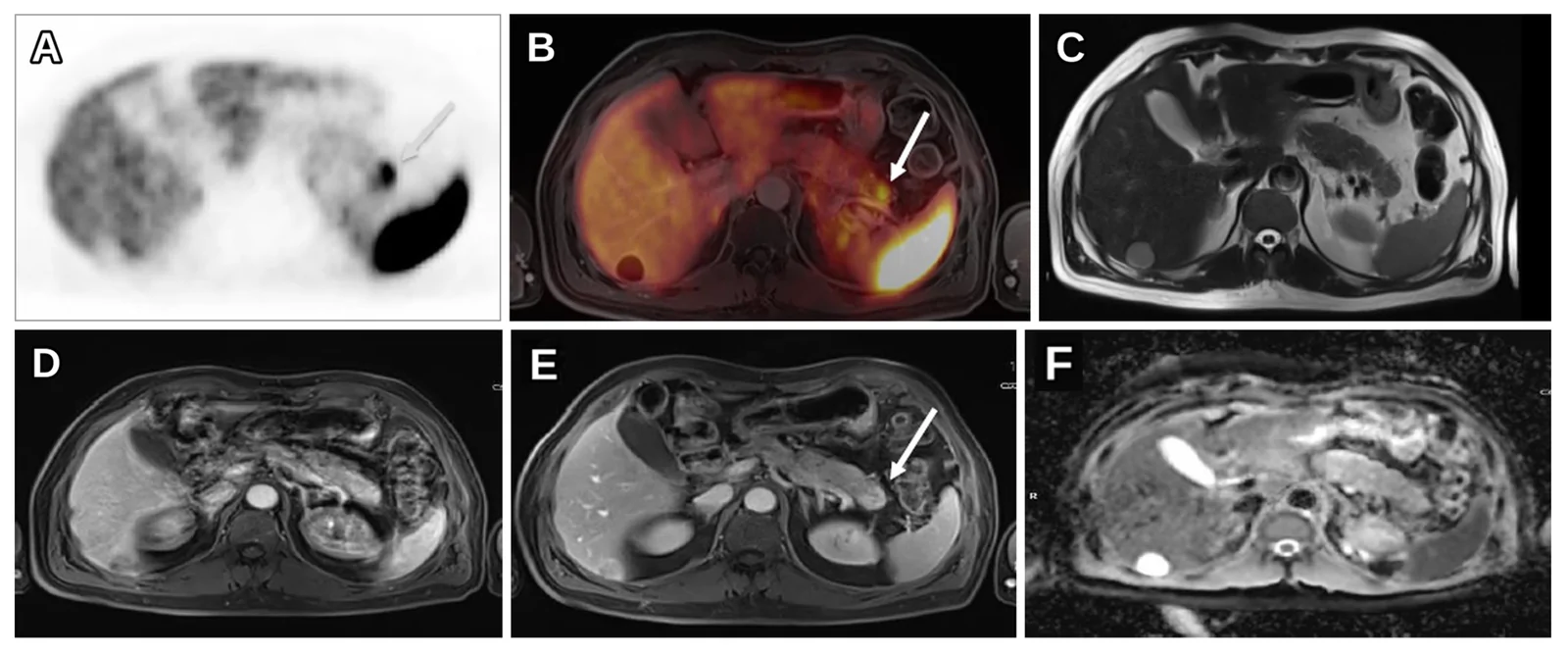
Initial staging with [^68^Ga]DOTATOC PET/MRI of a patient with a low-grade neuroendocrine tumor (NET) in the tail of the pancreas, diagnosed by echoendoscopy but not visualized on a previous contrast-enhanced CT scan. The lesion shows expression of somatostatin receptors on PET imaging and fuses with T1-weighted MRI ((**A**,**B**), arrow). It is not visualized on T2-weighted HASTE MRI (**C**). It is not detected on arterial-phase T1-weighted MRI (**D**) but is visible on venous-phase T1-weighted MRI ((**E**), arrow) and shows no abnormalities on diffusion-weighted imaging (DWI) MRI (**F**).

In the case of neuroendocrine tumors, PET/MRI using radiolabeled molecules targeting somatostatin receptors (DOTA-peptides) demonstrates improved detection of both the primary tumor and liver metastases compared to PET/CT (93.5% vs. 76.8%) [8,64] Figure 6.

### 4.6. Imaging and Radiomic Biomarkers

In pancreatic cancer, PET/MRI biomarkers such as those derived from diffusion sequences, spectroscopy, and metabolic volumes can predict clinical stage and progression-free survival [65]. Furthermore, distinctive radiomic features have been described in PET/MRI with the potential to differentiate the origin of liver metastases (pancreatic vs. gastrointestinal tumors) [66].

## 5. Brain Tumors

Regarding PET radiopharmaceuticals, given the limitations of [^18^F]FDG in assessing tumor viability in brain lesions—due to its high nonspecific brain activity—radiopharmaceuticals that assess amino acid metabolism, such as [^18^F]fluoroethyltyrosine (FET) and [^18^F]fluorodopa, or markers of membrane synthesis or lipid metabolism such as [^18^F]FCH [67,68,69,70] are used.

To determine the degree of response (pseudoprogression vs. progression), PET has advantages over MRI, as it is less influenced by post-treatment inflammatory phenomena. Therefore, in the presence of a negative or equivocal MRI and a positive PET, tumor viability will be suspected, whereas a suspicious MRI with a negative PET will be more indicative of treatment-related inflammatory changes [71].

**Figure 7:**
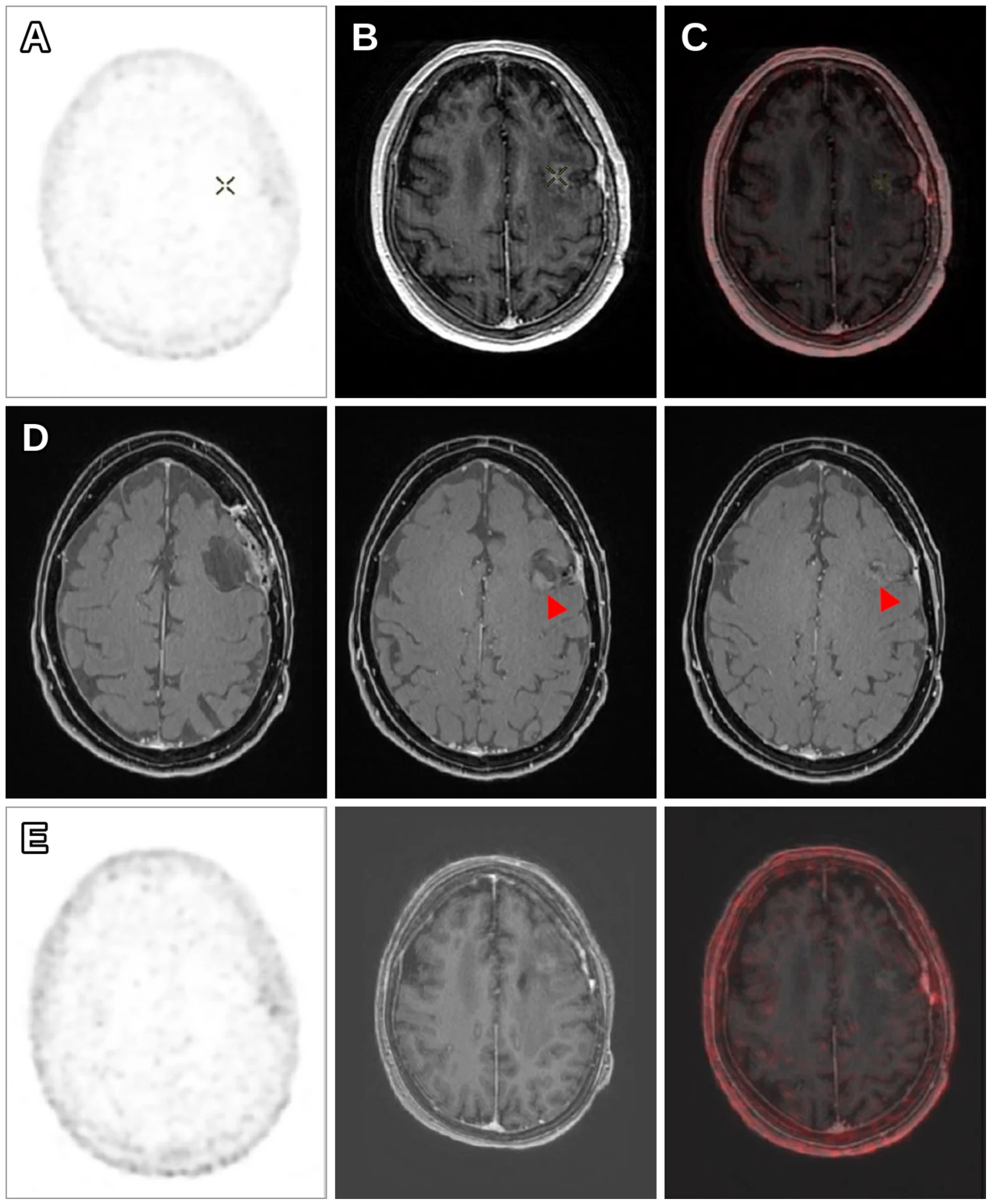
[^18^F]Fluorocholine PET/MRI of a post-surgery recurrence of an IDH wild-type left frontal glioblastoma. The patient underwent reoperation due to progression at the surgical site the same year. Eight months later, MRI showed an increase in VSCr at the posterior margin of the cavity, of uncertain significance; therefore, a PET/MRI scan with [^18^F]Fluorocholine was performed. (**A**) PET image showing no pathological uptake in the area identified as suspicious on MRI (cursor). (**B**) 3D T1-weighted reconstruction after intravenous contrast. (**C**) PET-MRI fusion (marked with a cursor: suspicious finding described on MRI). (**D**) Axial slices (cranial-caudal, left-right) of a fat-suppressed T1-weighted sequence highlighting the suspicious lesion (arrow head). [^18^F]Fluorocholine PET/MRI (**E**): the absence of uptake persists on the PET image, with stability on T1-weighted MRI after contrast, interpreted as coagulative necrosis following treatment with antiangiogenic agents.

**Figure 8:**
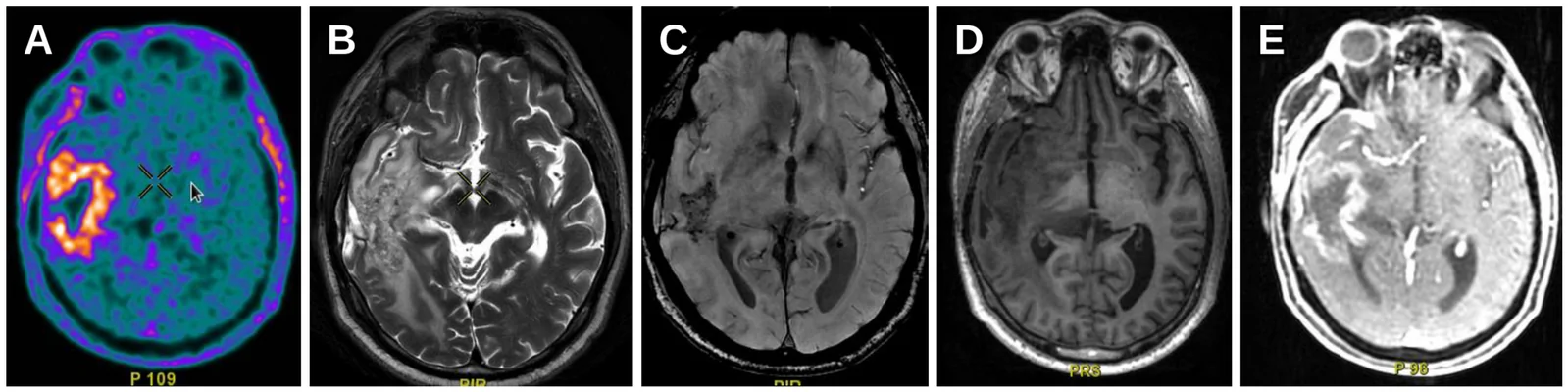
Post-treatment [^18^F]FET/MRI in a patient with a right temporal IDH wild-type glioma, who underwent surgery, temozolomide, and proton therapy: PET (**A**) and MRI sequences from (**B**–**E**): T2, SWAN (Susceptibility-Weighted Angiography), T1, and dynamic LAVA from a hybrid scan of a. The MRI performed prior to PET/MRI raised questions regarding the extent of tumor viability due to the extent of probable post-treatment changes. Irregular hyperuptake of [^18^F]FET (SUVmax 3.8/contralateral background SUVmean 0.7: TBR ratio 5.4) around the entire circumference of the right temporal resection margin, with a hypoactive center (**A**). MRI reveals postoperative changes with a gliotic cavity causing retraction of the temporal lobe. Increased T2 signal abnormality involving the posterior temporal region, anterior and posterior periinsular regions, and extending to the right frontal and parietal regions. Nodular enhancement around the entire circumference of the surgical resection margin (**E**).

**Figure 9:**
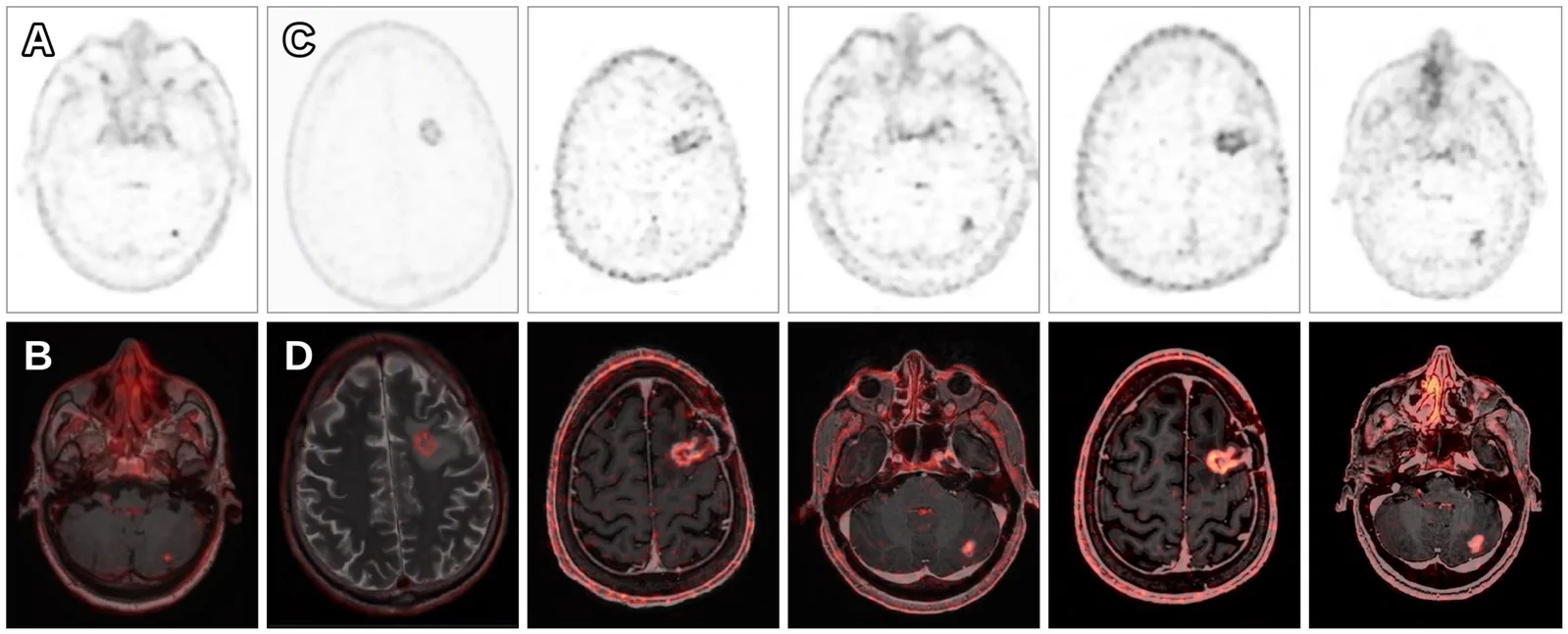
[^18^F]Fluorocholine PET/MRI of a patient who underwent surgery for a left frontal metastasis from lung adenocarcinoma and radiosurgery for a cerebellar metastasis detected only on [^18^F]Fluorocholine PET (**A**,**B**), PET and MRI co-registration, respectively), during follow-up. Subsequent MRI shows signs of radionecrosis in the frontal lesion bed and, less likely, tumor viability. Given the PET positivity at the site of the frontal lesion (**C**,**D**), PET and MRI co-registration, respectively), surgery was performed, revealing only necrotic tissue. PET/MRI reveals signs of viability both in the frontal lesion and in the bed of the treated cerebellar lesion (a new lesion), which shows signs of progression in subsequent studies. The patient continues to receive chemotherapy.

The use of hybrid imaging, which focuses primarily on primary tumors and is defined as the integrated acquisition and interpretation of PET/MRI, offers improved sensitivity in detecting tumor viability compared to PET/CT and MRI used in isolation (Figure 7 and Figure 8) [72]. In addition, although the evidence is more limited in other brain tumors, advantages may well be applicable to the context of metastatic disease (Figure 9).

In addition, the integrated acquisition and interpretation of PET/MRI offers improved molecular characterization of lesions, aiding in differential diagnosis, determining the degree of aggressiveness in heterogeneous lesions with predominantly low-grade features on previous imaging studies, or tumor locations with high surgical risk, or guiding biopsy, as described by Brendle et al. [73] using [^18^F]FET PET/MRI in 189 patients, with an 85% accuracy in identifying malignancy and a 33% change in therapeutic management.

However, the primary use of hybrid PET/MRI imaging focuses on the optimized differential diagnosis of recurrence/progression vs. radionecrosis, with a positive predictive value close to 100% and sensitivity and specificity both exceeding 90% [74,75], influencing clinical management in 53% of patients [73]. Furthermore, it has been shown to be a cost-effective tool in selected groups of patients with glial tumors [76].

### 5.1. Meningiomas

Initial results from hybrid PET/MRI imaging with DOTA-peptides indicate a promising role for these as imaging biomarkers, both in treatment planning and in assessing response in meningiomas treated with radiosurgery, while also demonstrating their cost-effectiveness [77,78,79].

Furthermore, in patients undergoing surgery, PET/MRI with DOTA-peptides increases diagnostic certainty in assessing the degree of tumor resection compared to MRI alone, allowing for more precise postoperative risk stratification and potentially avoiding unnecessary radiation therapy, thereby supporting its integration into postoperative decision-making for patients with World Health Organization grade 2 meningiomas [80].

### 5.2. Imaging Biomarkers and Radiomics

The integrated interpretation of radiomic variables from [^18^F]FET PET/MRI allows for more accurate genotypic classification than isolated assessment and even a more robust classification of IDH (isocitrate dehydrogenase) genotypes, suggesting a central role in the radiogenomic evaluation of diffuse gliomas prior to surgery [81,82].

The use of [^18^F]FCH PET/MRI has demonstrated its potential in determining tumor heterogeneity in gliomas and distinguishing post-treatment tumor viability, with particularly relevant advantages in posterior fossa tumors, where quantitative MRI alone may present greater technical challenges [83].

Representative semiquantitative PET tumor burden data, such as biological tumor volume, are presented as significant prognostic biomarkers in recurrent high-grade glioma, emphasizing tumor burden over metabolic activity or MRI perfusion [84].

## 6. Bone and Soft-Tissue Sarcomas

Sarcomas are tumors that present significant challenges in clinical and therapeutic management due to their considerable histological and biological heterogeneity [85]; therefore, imaging techniques guide surgical decisions and/or therapeutic strategies.

MRI is the gold standard for assessing the local staging of bone sarcomas (BS) and soft tissue sarcomas (STS), given its ability to accurately depict the tumor’s relationship with neurovascular structures, joints, and muscle compartments, as well as to evaluate bone marrow infiltration; thus, it is crucial for surgical and radiation therapy (RT) planning. The addition of new MRI techniques, such as diffusion-weighted imaging (DWI and ADC) and DCE studies, helps improve diagnostic accuracy [86].

[^18^F]FDG PET has high sensitivity in detecting distant metastases in high-grade sarcomas, in addition to enabling an effective assessment of metabolic response to neoadjuvant therapy and accurate classification of local recurrence [87,88,89]. However, the integrated assessment using [^18^F]FDG PET/MRI—and even more so its use as a multimodal technique—represents a major advance in the diagnostic management of this disease by combining PET’s high biological and molecular imaging accuracy with MRI’s superior soft-tissue contrast, enabling precise spatial and temporal co-registration. This facilitates the staging of both local and distant disease in a single test, shortening the diagnostic process while reducing radiation exposure—a factor of great importance in young and pediatric patients [90,91,92,93].

### 6.1. Tumor Characterization and Initial Staging

**Figure 10:**
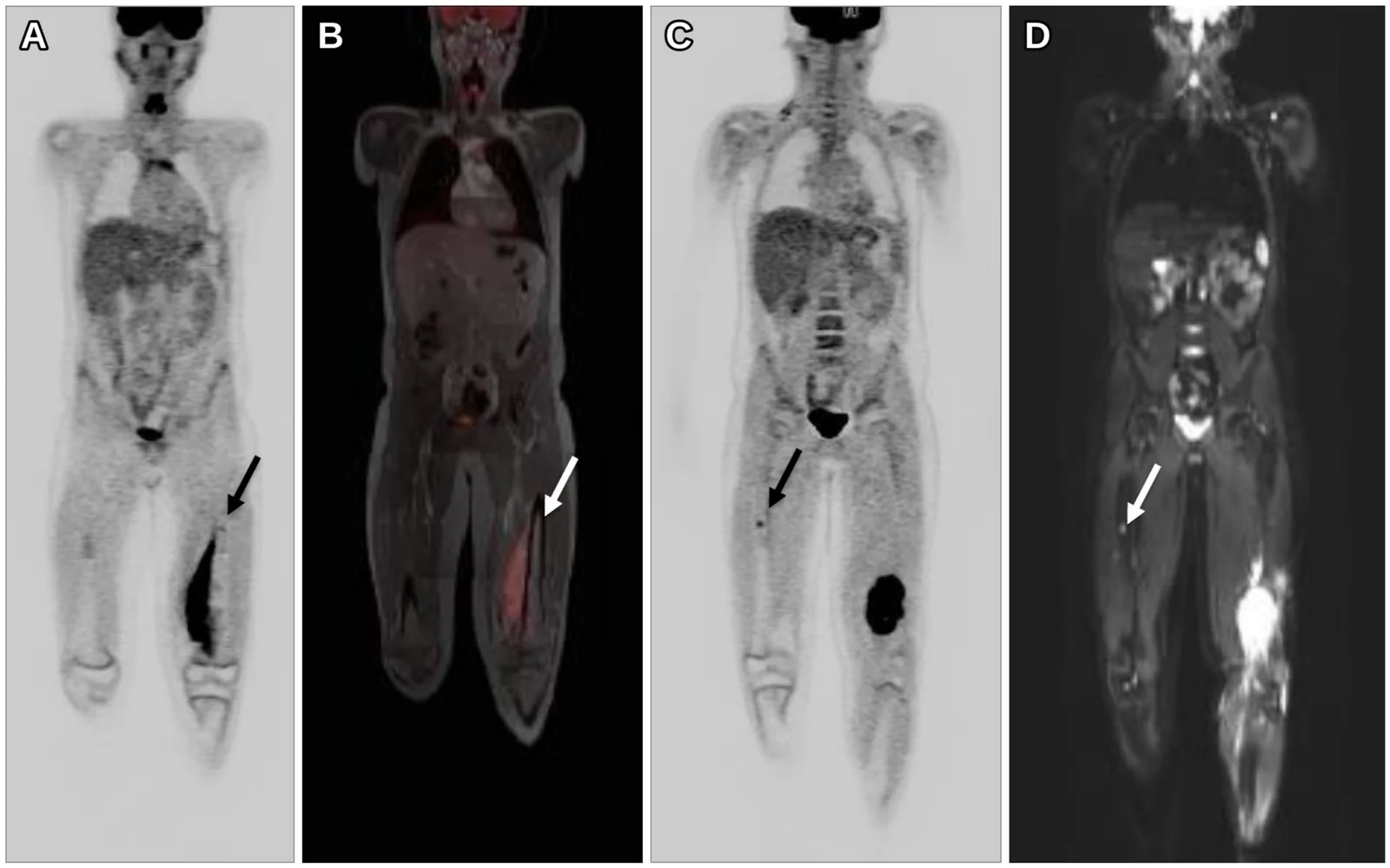
[^18^F]FDG PET/MRI of a patient with Ewing sarcoma of the left distal femur. ^18^Coronal sections: (**A**) PET; (**B**) PET fusion with T1 shows the primary tumor with intense uptake and skip metastasis (arrow) in the contralateral femur on PET (**C**) and DWI (diffusion-restricted, (**D**)), but it is not evaluable on the ADC map due to its small size. PET/MRI offers advantages in lymph node staging, demonstrating greater sensitivity for mediastinal and hilar lymphadenopathy compared to MRI alone, thanks to the integration of metabolic and anatomical information. Regarding non-rhabdomyosarcoma soft tissue sarcomas, the recommendations of the INSTRuCT International Consortium emphasize that PET/MRI may be useful in certain histological subtypes with a greater tendency toward lymph node metastasis, such as epithelioid sarcoma, angiosarcoma, or clear cell sarcoma [86]. However, the available evidence remains limited due to the rarity and heterogeneity of these tumors. Regarding the detection of small pulmonary metastases (<6 mm), high-resolution chest CT should remain a complementary modality in the staging protocol, given the limitations of MRI.

Tumor heterogeneity leads to extensive necrosis, intratumoral variability, and a risk of undersampling, which can result in errors in histological grading that could be reduced with the use of PET/MRI [89]. Therefore, the combined morphological and functional information from PET/MRI allows the biopsy to be targeted to the area of highest uptake (SUVmax) with low ADC, as this corresponds to high cellularity, thereby achieving greater diagnostic yield [94] Figure 10. Furthermore, it is an effective tool for defining surgical margins, more accurately identifying bone or neurovascular invasion, and reducing overestimation due to edema and inflammation compared to standalone MRI, thereby helping to reduce morbidity resulting from overtreatment (unnecessary amputations) [87,89,90,91].

The positive results of PET/MRI can be applied to the pediatric population [95,96,97,98,99]. European guidelines developed jointly by the European Pediatric Soft Tissue Sarcoma Study Group (EPSSG), the Cooperative Weichteilsarkom Studiengruppe, and the European Society of Pediatric Radiology recommend MRI as the primary technique for evaluating the primary tumor and locoregional spread, while PET/CT or PET/MRI is used to evaluate lymph nodes and extrapulmonary metastases [100].

### 6.2. Detection of Recurrence

Patients previously treated for sarcoma often have implanted metallic devices that can cause susceptibility artifacts due to metal interference, requiring the use of specialized imaging techniques to minimize the impact of these distortions on the detection of local recurrence [90].

Furthermore, assessment of the surgical bed presents additional challenges such as fibrosis, inflammatory changes, and post-radiotherapy edema. It is in this context that PET/MRI offers its greatest advantage by better differentiating between necrosis and viable tumor, given that malignant lesions generally have a higher SUVmax, greater volume, signal abnormalities, and enhancement compared to benign lesions. Therefore, hybrid imaging increases sensitivity and diagnostic confidence compared to MRI alone (90% vs. 83%, respectively), with better differentiation between necrosis and hematoma due to its superior soft-tissue resolution, thereby improving the delineation of malignant lesions compared to PET [101].

MRI has high sensitivity for detecting recurrence in pediatric patients, while PET with [^18^F]FDG can provide specificity when MRI findings are unclear, with the added advantage of lower exposure to ionizing radiation compared to PET/CT [102].

### 6.3. Response Assessment

The limitations of the RECIST (Response Evaluation Criteria in Solid Tumors) criteria include changes in size that do not reflect necrosis and post-treatment fibrosis, which mimics residual tumor; therefore, morphological assessment alone is insufficient. PET with [^18^F]FDG reveals tumor changes that are not detected by MRI alone [103,104]. Therefore, the integration of functional biomarkers from both techniques allows for combining their advantages (PET: early detection of metabolic changes compared to morphological changes; MRI: increased ADC or necrosis and reduced perfusion in the DCE study), thereby improving diagnostic reliability in response assessment [105].

In children, PET/MRI has been shown to be useful in assessing response to neoadjuvant therapy [92,93,106]. In this setting, European guidelines recommend continuing to use MRI for monitoring the primary tumor and using PET/CT or PET/MRI for the metabolic evaluation of lymph nodes and metastases. Metabolic response can be assessed using the Deauville scale or quantitative parameters such as SUV according to PERCIST (Positron Emission Tomography Response Criteria in Solid Tumors) criteria [104]. However, there is still insufficient evidence to use it as a prognostic biomarker capable of modifying treatment intensity [87].

### 6.4. Imaging and Radiomic Biomarkers

The combination of multiparametric MRI analysis with PET metabolic parameters such as SUV, MTV (metabolic tumor volume), and TLG (total lesion glycolysis) provides a powerful tissue characterization, enabling the determination of an integrated morphofunctional tumor phenotype, which also has prognostic implications [103,104,105,107].

## 7. Rectal Cancer

Multiparametric PET/MRI can improve identification of locally advanced rectal cancer (RC) and, hence, help in treatment stratification. In addition, interreader variability is lower between readers assessing PET/MRI compared with MRI alone [108].

MRI is the gold standard for T- and N staging in RC [109]. Current baseline nodal criteria from the 2016 European Society of Gastrointestinal and Abdominal Radiology (ESGAR) rely on size and morphological features with methodological limitations affecting reliability [110,111,112].

### 7.1. Initial Staging

Extended or 15-min PET acquisition in the pelvis detects 40% more [^18^F]FDG-avid lymph nodes than a standard 3-min PET acquisition [108].

Compared to MRI alone, [^18^F]FDG PET/MRI offers potential added value by reducing the risk of nodal understaging. Combined [^18^F]FDG PET/MRI criteria for malignancy resulted in a sensitivity of 76% and a specificity of 90% [113].

Regarding distant metastatic disease detection, in a recent review, including 22 observational studies, PET/MRI proved superior, leading to as much as a 21% change in management [114].

### 7.2. Response to Neoadjuvant Treatment

In RC, major or complete clinical response following neoadjuvant ChRT is observed in 10–60% of patients depending on the neoadjuvant treatment (NT) regimen used and initial tumor stage [115].

Nonoperative management (NOM) is an increasingly utilized treatment strategy for selected patients who achieve a clinical complete response (cCR) after NT, with low rates of local recurrence at 12 months post-treatment. Maximizing imaging sensitivity for residual or recurrent disease is critical to ensuring that cCR is accurately identified and that NOM failures are detected early, when surgery is still feasible and potentially curative.

Despite the high accuracy of pelvic MRI for initial staging, its ability to predict the presence and extent of tumor following neoadjuvant ChRT is limited, with a mean overall sensitivity of only 40.3% for tumor response [116,117].

The hybrid PET/MRI approach potentially overcomes the aforementioned limitations seen in MRI and PET/CT. [^18^F]FDG might be complementary to MRI for treatment response assessment, detecting more residual disease after TNT than MRI alone, with the [^18^F]FDG PET component providing added value in most restaging/surveillance scans [118,119]. Using hybrid imaging, PET/MRI demonstrated sensitivity, specificity, and accuracy values of 0.88, 0.80, and 0.84, respectively, for detecting pCR, compared to 0.82, 0.50, and 0.67 for standard MRI. PET/MRI accurately identified tumor regression grade stages in 72% of cases, compared to 50% for standard MRI. Post-SUV, post-ADC, and delta-ADC were the most precise PET/MRI predictors for pCR, with AUC values of 0.81, 0.75, and 0.55, respectively [120].

### 7.3. Recurrence Detection

[^18^F]FDG PET/CT and whole-body PET are valuable imaging tools for detecting and localizing colorectal cancer recurrence in the presence of elevated carcinoembryonic antigen levels [121]. Few studies using PET/MRI have reported sensitivity and specificity of 94%, with better accuracy and confidence level of readers, reducing the number of equivocal MRI findings (5% vs. 12%) [122,123,124].

### 7.4. Imaging and Radiomic Biomarkers

Multiparametric PET/MRI provides additional information on RC tumor biology and may have prognostic value [125]. Locally advanced rectal cancers exhibit significantly higher MTV and TLG compared to early tumors. MTV has been independently associated with overall survival.

Regarding biological tumor characteristics, PET/MRI can be used to differentiate the mucinous and nonmucinous components within primary RC based on metabolic status. SUVmean is significantly lower in tumors with a mucinous component than in those without a mucinous component, whereas tumor ADC measurements are not different between tumors with and those without a mucinous component. Thus, [^18^F]FDG uptake is significantly lower in the mucinous component, but tumor cellularity based on MRI and DWI findings is not [126]. In addition, ADC mean values are correlated with the tumor stroma ratio (TSR), and stroma-high (low TSR) rectal cancer corresponded to relatively lower ADC mean values. The ADC mean and ADC minimum (ADCmin) values were found to be negatively correlated with the pathological T stages as well as the largest tumor diameters of RC. In addition, the pathological T stages correlated with all PET-related metabolic parameters: SUV mean, SUVmax, MTV, and TLG. However, there were no statistical correlations between the PET/MRI parameters and the immunohistochemical results [127].

## 8. Breast Cancer

In patients with breast cancer, the integrated use of molecular and multiparametric imaging focuses on a limited group of patients—specifically those at highest risk for metastatic disease (where both imaging modalities play complementary roles) and in the characterization of locoregional post-treatment findings that are inconclusive with CT.

### 8.1. Detection of Primary Tumors

MRI has very high sensitivity for detecting breast cancer, assessing local tumor spread, and identifying occult breast lesions, although it has moderate specificity. The combination of PET/MRI increases diagnostic accuracy compared to either technique alone, with advantages in detecting multifocality and multicentricity [128]. The sensitivity, specificity, and area under the curve (AUC) of [^18^F]FDG-PET/MRI for tumor detection are 95%, 91%, and 0.96, respectively [129]. Furthermore, selective imaging in the prone position is superior to whole-body imaging in the supine position for the accurate identification of breast cancer (97% and 87%, respectively) [130].

### 8.2. Detection of Regional and Distant Disease

**Figure 11:**
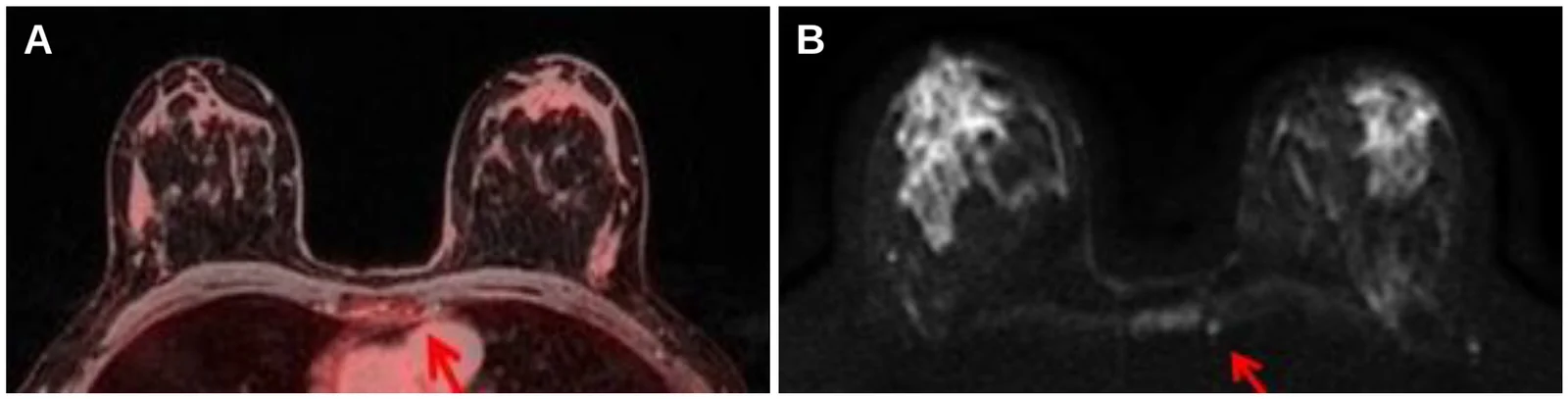
Dynamic post-contrast [^18^F]FDG PET/T1 MRI (**A**) of a patient with breast cancer showing increased metabolism over a millimeter-sized lymph node in the right internal mammary region (arrow) with diffusion restriction (**B**). Furthermore, the detection of regional and/or distant disease using [^18^F]FDG-PET/MRI may lead to changes in treatment management in up to 33% and 12% of patients, respectively, compared with CT and [^18^F]FDG-PET/CT with contrast [128,135,136,137].

[^18^F]FDG-PET/MRI provides a better assessment of the axillary region compared to CIT, increasing diagnostic confidence in the detection of lymph node involvement based on [^18^F]FDG uptake on PET and diffusion restriction on MRI. A meta-analysis including 4 studies showed sensitivity, specificity, and AUC of 94%, 90%, and 0.96, respectively [131]. Morawitz et al. [132] demonstrated a higher number of regionally infiltrated lymph nodes on [^18^F]FDG-PET/MRI compared with MRI and CT (41.2%, 30.8%, and 20.8%, respectively). Furthermore, PET can enhance the sensitivity of MRI, especially for millimeter-sized lesions, and its combined use is particularly relevant for detecting extra-axillary disease. In this regard, García et al. [133], in a prospective study, reported infiltration of the internal mammary chain in 34% of patients with stage ≥ IIB, enabling personalized therapies, with the implementation of thoracic radiation therapy in 24% of patients (Figure 11). However, clinical trials evaluating the reliability of [^18^F]FDG-PET/MRI with a view to replacing sentinel lymph node biopsy (SLNB)—currently considered the gold standard—in axillary staging are still pending [134].

[^18^F]FDG-PET/MRI is more accurate than CIT, including PET/CT, in detecting bone and liver metastases [138,139]. de Mooij et al. [140], in a systematic review, demonstrated greater sensitivity in detecting metastatic lesions with [^18^F]FDG-PET/MRI compared with [^18^F]FDG-PET/CT (89% vs. 77%), distinguishing between liver metastases (80–100% vs. 70–75%) and bone metastases (92–98% vs. 69–99%). Another meta-analysis (3 studies, 182 patients) reported higher sensitivity and specificity for [^18^F]FDG-PET/MRI compared with [^18^F]FDG-PET/CT: 95% versus 87% and 96% versus 94%, respectively [141]. The superior performance of whole-body PET/MRI staging is explained by MRI’s greater sensitivity in detecting lesions that do not take up [^18^F]FDG (permeative bone metastases and subcentimeter liver metastases) [142].

Therefore, when available, the use of [^18^F]FDG-PET/MRI for staging patients with locally advanced invasive breast cancer or high-risk cases would be justified, as it shares the same clinical indications as [^18^F]FDG-PET/CT, with the added advantage of exposing patients to a lower radiation dose [143].

### 8.3. Response to Neoadjuvant Chemotherapy

When comparing PET with MRI in the early prediction of pathological complete response (pCR), PET is more sensitive, and MRI is more specific [144]. Therefore, [^18^F]FDG-PET/MRI may be recommended as the preferred single-step technique, as it seems to offer the highest diagnostic yield in the noninvasive prediction of pCR [145].

### 8.4. Recurrence Detection

Regarding the detection of recurrence, no major differences have been reported between [^18^F]FDG-PET/MRI and PET/CT in detecting recurrent disease, whether analyzed by lesion or by patient [146].

### 8.5. Imaging and Radiomic Biomarkers

Some studies, focused on evaluating the role of PET and MRI variables obtained from hybrid [^18^F]FDG-PET/MRI scans in predicting axillary lymph node metastases, have reported significant accuracy using machine learning-based models [147,148]. However, the potential of radiomic variables obtained from baseline [^18^F]FDG-PET/MRI in predicting response to neoadjuvant therapy, particularly in patients who are hormone receptor-positive and HER2-negative, needs deep investigation; this technology can be classified as emerging [149,150].

## 9. Conclusions

Since PET-MRI is not currently a technique recommended in clinical guidelines, the recommendations issued are based on the experience of the centers, supported in some cases by evidence reported in various clinical studies.

We proposed, based on our experience and the current literature, the recommendations summarized in Table 1. Regarding the limited evidence, the strength to support these recommendations is low. Thus, both for diagnostic and radiomic purposes, prospective multicenter studies are necessary for validation before clinical implementation.

As availability of PET-MRI increases and technological limitations decrease, PET/MRI is expected to play an increasingly important role in the management of cancer patients, particularly within the context of personalized medicine and curative therapeutic strategies.

## Figures and Tables

**Table 1:** Potential recommendations of PET/MRI in Oncological processes.

Tumor Type	Applications
Prostate Cancer	Biopsy guidance in patients with an intraprostatic lesion and a previous negative biopsy. Characterization of lesions classified as indeterminate on MRI (PI-RADS 3). Initial staging in high-risk patients who are candidates for curative local therapies. Restaging due to biochemical recurrence in high-risk patients who are candidates for local treatments.
Gynecologic Tumors	Staging of inoperable cervical cancer (from IB3) according to FIGO, prior to selection of local treatments. Guidelines for radiation therapy planning in cervical cancer. Assessment of response to ChRT in cervical cancer. Detection of recurrence of gynecologic tumors in cases of uncertainty regarding CIT in patients who are candidates for salvage therapy. Assessment of peritoneal carcinomatosis in patients eligible for salvage therapy with CIT.
HBP Tumors	Staging or restaging of primary tumors with liver involvement in patients who are candidates for surgery or transplantation due to CIT.
Brain Tumors	Biopsy guidelines for patients with low-grade tumors on MRI showing signs suggestive of high-grade disease (applicable to gliomas and meningiomas). Follow-up during or after the Stupp protocol in patients with high-grade glioma. Determining whether post-treatment changes or active disease are present in any type of tumor (primary or metastatic). Guidelines for radiation therapy planning.
Bone and Soft-Tissue Sarcoma	Characterization and biopsy guidelines for tumors of uncertain classification based on CT or even prior MRI, especially if a previous biopsy was inconclusive or inconsistent with the clinical suspicion. Staging in patients with high-grade tumors prior to debulking surgery. Suspected local or distant recurrence based on previous CT scans. Assessment of response to neoadjuvant therapy. Guidance for radiation therapy planning.
Rectal Cancer	Initial staging in patients with locally advanced tumors based on CT imaging, who are candidates for neoadjuvant therapy or resection surgery. Assessment of response to neoadjuvant therapy for surgical planning. Detection of recurrence in patients with inconclusive CT findings who are candidates for local salvage therapy.
Breast Cancer	Initial staging in patients with locally advanced tumors detected by CT, who are candidates for neoadjuvant therapy or resection surgery. Assessment of response to neoadjuvant therapy for surgical planning. Detection of recurrence in patients with inconclusive CIT results who are candidates for local salvage therapy.

HBP: hepatobiliary-pancreatic; PI-RADS: Prostate Imaging Reporting and Data System; FIGO: International Federation of Gynecology and Obstetrics; ChRT: chemoradiotherapy; CIT: conventional imaging techniques.

## Data Availability

All data generated or analyzed during this study are included in this published article.
